# Mycophenolic Acid Inhibits Migration and Invasion of Gastric Cancer Cells via Multiple Molecular Pathways

**DOI:** 10.1371/journal.pone.0081702

**Published:** 2013-11-15

**Authors:** Boying Dun, Ashok Sharma, Yong Teng, Haitao Liu, Sharad Purohit, Heng Xu, Lingwen Zeng, Jin-Xiong She

**Affiliations:** 1 Guangzhou Institutes of Biomedicine and Health, Chinese Academy of Sciences, Guangzhou, China; 2 Institute of Translational Medicine, School of Pharmaceutical Sciences, Nanjing University of Technology, Nanjing, China; 3 Center for Biotechnology and Genomic Medicine, Medical College of Georgia, Georgia Regents University, Augusta, Georgia, United States of America; 4 Cancer Center, Georgia Regents University, Augusta, Georgia, United States of America; University of Georgia, United States of America

## Abstract

Mycophenolic acid (MPA) is the metabolized product and active element of mycophenolate mofetil (MMF) that has been widely used for the prevention of acute graft rejection. MPA potently inhibits inosine monophosphate dehydrogenase (IMPDH) that is up-regulated in many tumors and MPA is known to inhibit cancer cell proliferation as well as fibroblast and endothelial cell migration. In this study, we demonstrated for the first time MPA’s antimigratory and anti-invasion abilities of MPA-sensitive AGS (gastric cancer) cells. Genome-wide expression analyses using Illumina whole genome microarrays identified 50 genes with ≥2 fold changes and 15 genes with > 4 fold alterations and multiple molecular pathways implicated in cell migration. Real-time RT-PCR analyses of selected genes also confirmed the expression differences. Furthermore, targeted proteomic analyses identified several proteins altered by MPA treatment. Our results indicate that MPA modulates gastric cancer cell migration through down-regulation of a large number of genes (*PRKCA*, *DOCK1*, *INF2, HSPA5, LRP8* and *PDGFRA*) and proteins (PRKCA, AKT, SRC, CD147 and MMP1) with promigratory functions as well as up-regulation of a number of genes with antimigratory functions (*ATF3*, *SMAD3*, *CITED2* and *CEAMCAM1*). However, a few genes that may promote migration (*CYR61* and *NOS3*) were up-regulated. Therefore, MPA’s overall antimigratory role on cancer cells reflects a balance between promigratory and antimigratory signals influenced by MPA treatment.

## Introduction

Most solid tumors are largely curable if they are diagnosed and treated before dissemination beyond the initial primary site. However, once tumors spread to other locations, they usually become highly morbid and likely fetal. Therefore, it is essential to limit the initial dissemination and prevent secondary spreading. Invasion and metastasis are two major modes of tumor dissemination, each driven by different mechanisms and leading to distinct therapeutic challenges[1]. However, both forms of dissemination require cell movement from the primary sites to the secondary location as well as reestablishment and survival of the tumor cells in the secondary locations. These processes are accomplished via a cascade of molecular changes within the cancer cells. Although the molecular mechanism underlying cell migration has been extensively studied, novel insights into the molecular events may provide new therapeutic approaches. Indeed, inhibition of the molecules that promote cell migration and up-regulation of molecules that inhibit cell migration through pharmacological interventions have become an important part of the weaponries to combat cancer. Development or repurposing of additional drugs that inhibit migration and invasion is a major ongoing effort for cancer therapeutics.

Mycophenolate mofetil (MMF) has been approved for the prevention of acute graft rejection in kidney, heart, and liver transplantation [2,3]. MMF is the morpholinoethyl ester prodrug of mycophenolic acid (MPA), which is a potent uncompetitive inhibitor of inosine monophosphate dehydrogenase (IMPDH), the rate-limiting enzyme for the *de novo* synthesis of guanosine nucleotides [4,5], which play crucial roles in cell proliferation and other cellular functions including DNA replication, RNA and protein synthesis and cellular signaling [6]. Consequently, MPA blocks T and B lymphocyte proliferation and clonal expansion, and prevents the generation of cytotoxic T cells and other effector T cells. Other mechanisms may also contribute to the efficacy of MPA in preventing allograft rejection. Through depletion of guanosine nucleotides, MPA can suppress glycosylation and the expression of several adhesion molecules, thereby decreasing the recruitment of lymphocytes and monocytes into sites of inflammation and graft rejection [5]. 

 Since IMPDH expression is significantly up-regulated in many tumor cells [7,8], it is, therefore, potentially a target for cancer therapy in addition to immunosuppressive chemotherapy. MPA/MMF has been reported to inhibit cancer cell proliferation and induces apoptosis in many cancer cells [9–14] . MPA/MMF has also been reported to inhibit migration of fibroblast cells [15] and human umbilical vein endothelial cells (HUVECs) [16]. However, it is unknown whether MPA can alter the migration and invasion capacity of cancer cells. Furthermore, the precise migration signaling pathways and effector molecules underlying MPA’s activities remain elusive. 

In this study, we first demonstrated that MPA significantly changes the migration and invasion ability of AGS cells and we then used gene expression and proteomic technologies to identify genes and proteins underlying these functions.

## Materials and Methods

### Cell lines, reagents and antibodies

Two gastric cancer cell lines (AGS and Hs746T) were obtained from the American Type Culture Collection (ATCC). Both cell lines were grown in RPMI 1640 medium containing 10% fetal bovine serum, 100 units/ml of penicillin and 100µg/ml of streptomycin at 37°C with 5% CO2. MPA was purchased from VWR. The CD147, the integrin beta5 antibody was purchased from Abcam, the GAPDH and ICAM-1antibodies from Santa Cruz; Src, Akt, and p-Akt (Ser473) antibodies from Cell Signaling. 

### In vitro trans-well migration and invasion assays

Cell migration was performed with the Transwell (Costar) system, which allows cells to migrate through 8-μm pore size polycarbonate membrane. In brief, the serum starved AGS or HS746T cells were added to the upper chamber (5×104 cells per insert) and DMEM medium with different concentration of MPA (1µg/ml, 1.5µg/ml and 2µg/ml) was used as a chemoattractant in the lower chamber. After incubation at 37°C for 8 hours, the cells in the lower chamber were fixed in methanol and stained with 0.2% crystal violet. Numbers of the migrating cells in nine randomly selected fields from triplicate chambers were counted in each experiment under a phase-contrast microscope. The invasive potential of the cells was analyzed using a Matrigel-coated modified Boyden chamber (BD biosciences, San Jose, CA, USA) as described previously [17]. DMEM containing MPA was added to the lower chamber. After incubation at 37°C for 24 hours, the number of cells that invaded to the lower side of the upper chamber was counted.

### Micorarray experiments

Total RNA was extracted from AGS cells using a magnatic beads RNA extraction kit (Jinfiniti Biosciences, Augusta, GA). Gene expression profiling was performed using the human Illumina HumanHT-12 v4 BeadChip (Illumina, San Diego, CA). An aliquot of 200ng of total RNA was converted into double stranded cDNA (ds-cDNA) by using the Illumina TargetAmp-Nano labeling kit with an oligo-dT primer containing a T7 RNA polymerase promoter (Genset, St. Louis, MO). *In vitro* transcription was performed on the above ds-cDNA using the Enzo RNA transcript labeling kit. Biotin-labeled cRNA was purified by using an RNeasy affinity column (Qiagen), and fragmented randomly to sizes ranging from 35-200 bases by incubating at 94°C for 35 min. The hybridization solutions contained 100mM MES, 1 M Na^+^, 20 mM EDTA, and 0.01% Tween 20. The final concentration of fragmented cRNA was 0.05 µg/µl in hybridization solution. Target for hybridization was prepared by combining 40 µl of fragmented transcript with sonicated herring sperm DNA (0.1 mg/ml), BSA and 5nM control oligonucleotide in a buffer containing 1.0 M NaCl, 10 mM Tris.HCl (pH7.6), and 0.005% Triton X-100. Target was hybridized for 16h at 45°C in an Illumina hybridization oven. Chips were then washed at 50°C with stringent solution, then again at 30°C with non-stringent washes. Arrays were then stained with streptavidin-Cy3. The microarray data are MIAME compliant and have been deposited in NCBI Gene Expression Omnibus and are accessible through GEO Series accession number GSE46671.

### Real-time RT-PCR analysis

An aliquot of total RNA (2µg per sample) were arrayed in 96-well plates and then converted to cDNA using a High Capacity cDNA Reverse Transcription Kit (Applied Biosystems) and a PTC-100TM programmable thermal controller (MJ Research, Inc). The cDNA products were used for quantitative real-time PCR using ready-to-use TaqMan gene expression assays from the Applied Biosystems. Five reference genes with relatively constant expression (GAPDH, ESD, GUSB, IPO8 and MRPL19) were used for normalizing RNA concentration. Real-time PCR was performed with 96x96 Fluidigm Expression chips. Standard thermal cycling condition (10 min at 95°C, 40 cycles for 15 sec at 95°C, 1 min at 60°C) was used for all genes. All samples were analyzed on the same plate and each sample was analyzed in duplicate. 

### Western blotting

Cells were harvested and resuspended in PBS. After centrifugation at 2000rpm for 5 min, the pellet was lysed in ice cold M-PER Mammalian Protein Extraction Reagent containing 1% Halt Protease and Phosphatase Inhibitor Cocktail (Thermo Scientific) for 30 min. The supernatant was collected after 10 min of centrifugation at 12000rpm, equaled by spectrophotometry, denatured with sample loading buffer for 10 min at 95ᵒC and stored at 4°C for future use. Proteins were separated by 10% SDS-polyacrylamide gels and transferred to PVDF membrane (Bio-Rad), and incubated with primary antibodies of interest at 4°C overnight. The appropriate horseradish-conjugated secondary antibody at a dilution of 1:10000 in blocking buffer (3% BSA-TBST) was added and incubated for 1h at room temperature. All antibodies were diluted in 3% BSA-TBST. Immunoblots were developed using ECL chemiluminescence reagent (Thermo Scientific).

### Flow cytometry

Cells were trypsinized and washed twice in PBS. Approximately 1x10^6^ cells were incubated with 1µg of anti-CD147, anti-ICAM-1 and anti-integrin beta 5 antibodies for 30min, washed with PBST twice, then incubated with a secondary antibody conjugated with TRITC or APC for 30min, and washed twice with PBST. Flow cytometric analyses were performed on a FACScaliburTM flow cytometer with CELLQuestTM software (Becton Dickinson, Franklin Lakes, NJ, USA).

### Luminex assay

MMP proteins in culture medium were measured using Luminex kits from Millipore Company (Billerica, MA, USA) according to manufacturer’s recommendation. Cell culture medium (1:10 dilution) was incubated with the antibody-coupled microspheres and then with biotinylated detection antibody before the addition of streptavidin-phycoerythrin. The captured bead-complexes were measured with the FLEXMAP 3D system (Luminex, Austin, TX). Median fluorescence intensity (MFI) was collected and used for calculating protein concentrations.

### Statistical analysis

All statistical analyses were performed using the R language and environment for statistical computing (www.r-project.org). The comparisons between group means were made by ANOVA (for ≥ 3 groups) followed by pair-wise comparisons using Bonferroni post-hoc testing.  The statistical significance of differences was set at P < 0.05. 

The lumi package was used to preprocess microarray data. Differential expression analyses were conducted using the limma package from the Bioconductor project [18]. We used the false discovery rate (FDR) to adjust for multiple testing [19]. A combination of adjusted p-value and absolute value of fold change (FC) were used for selecting the differentially expressed genes. 

Cluster analysis was performed for grouping differentially expressed genes exhibiting similar expression patterns using the HPCluster program [20]. The Bioconductor package “GOstats” was used for testing the association of Gene Ontology terms (biological processes) to the differentially expressed genes [21] A p-value based on the hypergeometric test was computed to assess whether the number of genes associated with the term is larger than expected. The p-values obtained were adjusted for multiple testing using the FDR. The gene list was also analyzed using prebuild KEGG pathways. We used the “spia” (signaling pathway impact analysis) package to identify pathways impacted by the observed changes in the gene expression [22]. Network analysis was performed to construct molecular interaction networks using the STRING database [23]. The networks were imported in simple interaction format to Cytoscape for visualization [24]. 

## Results

### MPA inhibits AGS cell migration and invasion

**Figure 1:**
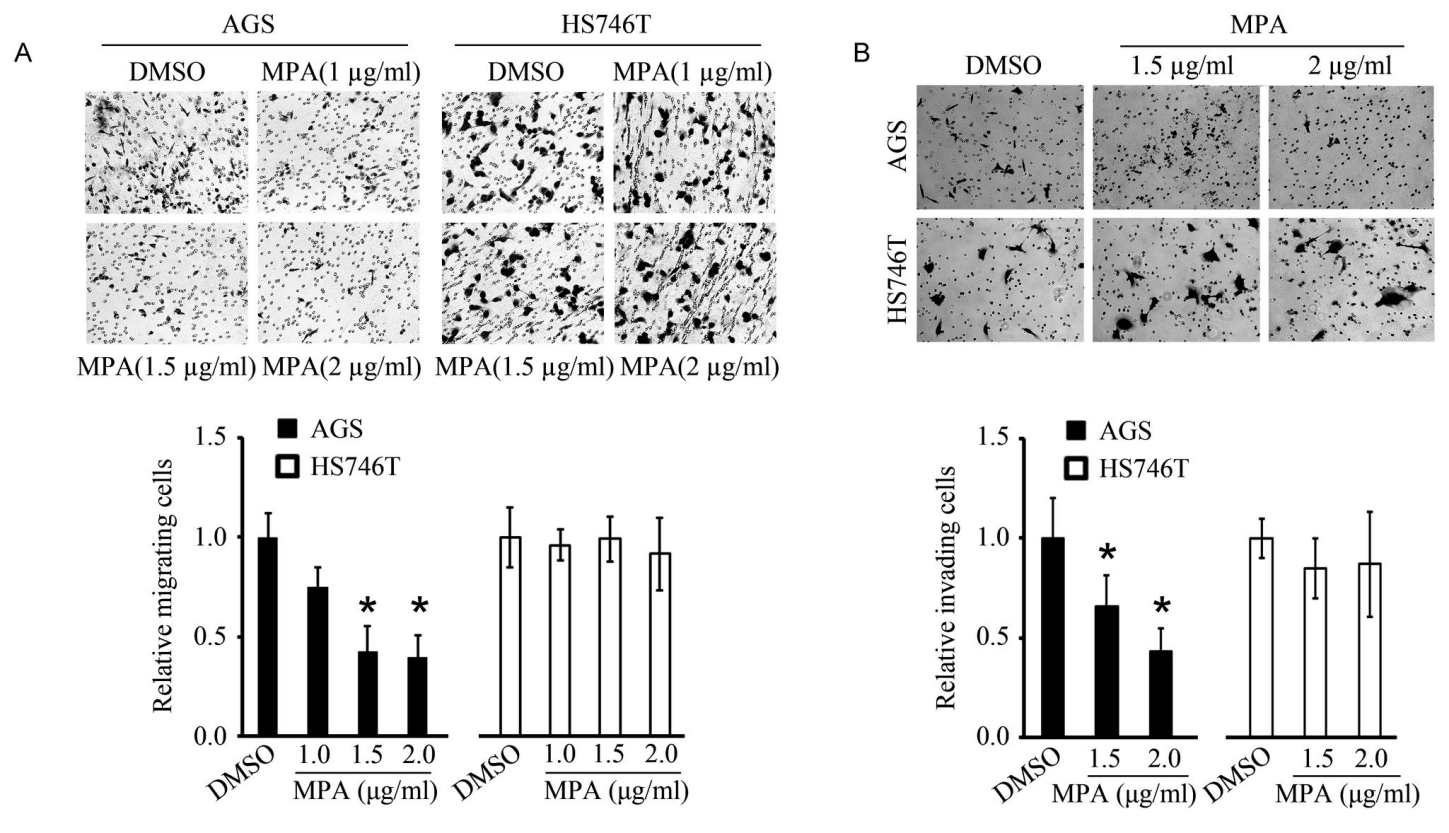
MPA modulates the migration and invasion abilities of AGS cells but not Hs746T cells. **A**: Images of migrating cells after treatment with vehicle (DMSO) or different concentration of MPA for 24h. The mean relative migrating cells are shown at the bottom. **B**: Images of invading cells after treatment with vehicle (DMSO) or different concentration of MPA for 24h. The mean relative invading cells are shown at the bottom. *p < 0.05 compared to DMSO control.

The migratory ability of two gastric cancer cell lines (AGS and Hs746T) with and without MPA treatment was assessed using 8µm pore transwell chambers without matrigel. As shown in Figure 1A, the percentages of migrating AGS cells decreased in a MPA dose-dependent manner, reducing to less than 50% of the control AGS cells at concentrations of >1.5µg/ml. However, the migratory ability of Hs746T cells was not affected by MPA at similar concentrations. Similarly, cell invasion was assayed using 8µm pore Biocoat Matrigel 8-micron invasion chambers (Figure 1B). MPA treatment also significantly decreased the invading ability of AGS cells but not Hs746T cells.

### MPA-induced gene expression changes related to cancer cell migration

To identify the genes altered by MPA treatment, we conducted a global transcriptomic profiling experiment with AGS cells treated with MPA. Using the entire gene expression dataset, we identified KEGG pathways that are significantly altered by MPA. Eight of the top ten significantly changed pathways (p53 signaling, cell cycle, pathways in cancer, PPAR signaling, bladder cancer, protein processing in ER, small cell lung cancer and MAPK signaling) are well-known to be cancer-related. The differentially expressed genes were also mapped to Gene Ontology biological processes and a hypergeometric test was performed to evaluate the significance of enrichment for each category. Our analysis indicated that the significantly enriched genes are implicated in cell cycle (1.6 fold enrichment, p < 10^-5^), cell death (1.7-fold enrichment, p < 10^-7^), cell proliferation (1.8-fold enrichment, p < 10^-7^), and cell migration (1.5 fold enrichment, p < 0.005). The global gene expression data are consistent with MPA’s activity in inhibiting cancer cell proliferation, induction of apoptosis and inhibition of migration. 

**Figure 2:**
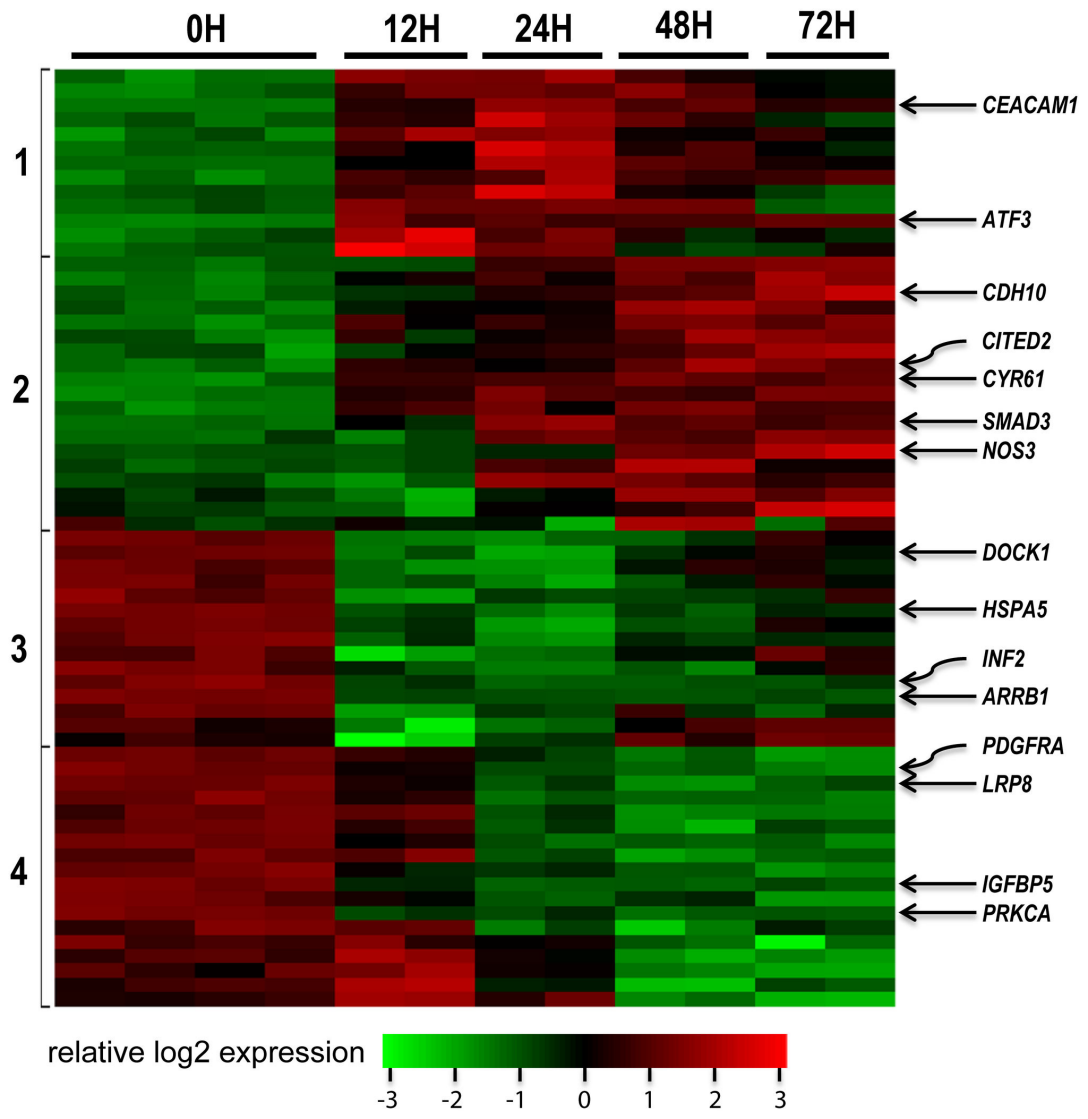
Molecular changes related to migration. Heatmap for genes differentially expressed after MPA treatment for 0h, 12h, 24h, 48h and 72h. Each sample is represented in a column and each gene is represented in a row. Increased expression is indicated as red and decreased expression is indicated as green. Representative genes are shown on the right panel and gene clusters are indicated on the left.

**Figure 3:**
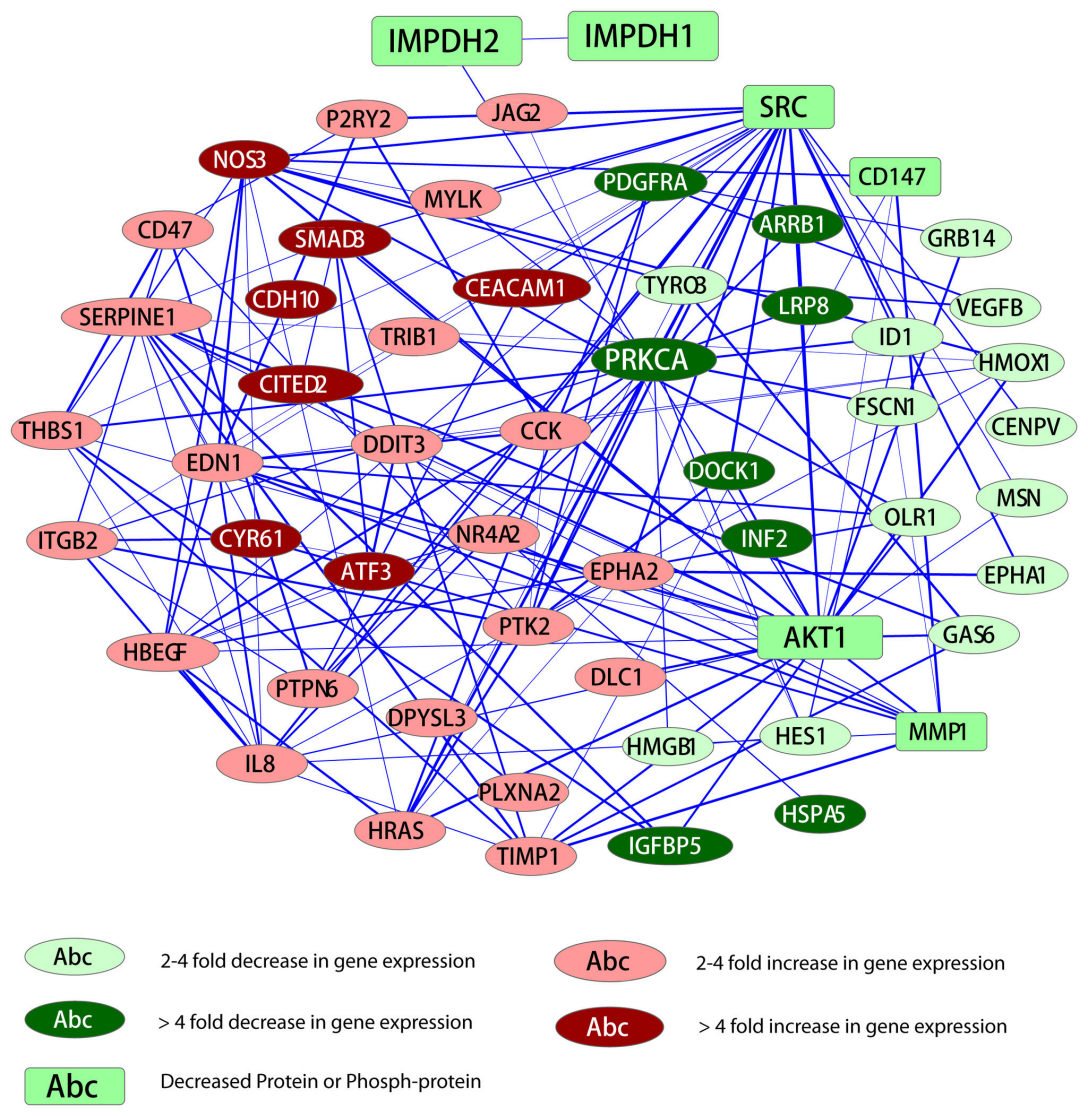
A network illustrating the connectedness of the genes (>2-fold differences) involved in cell migration.

**Figure 4:**
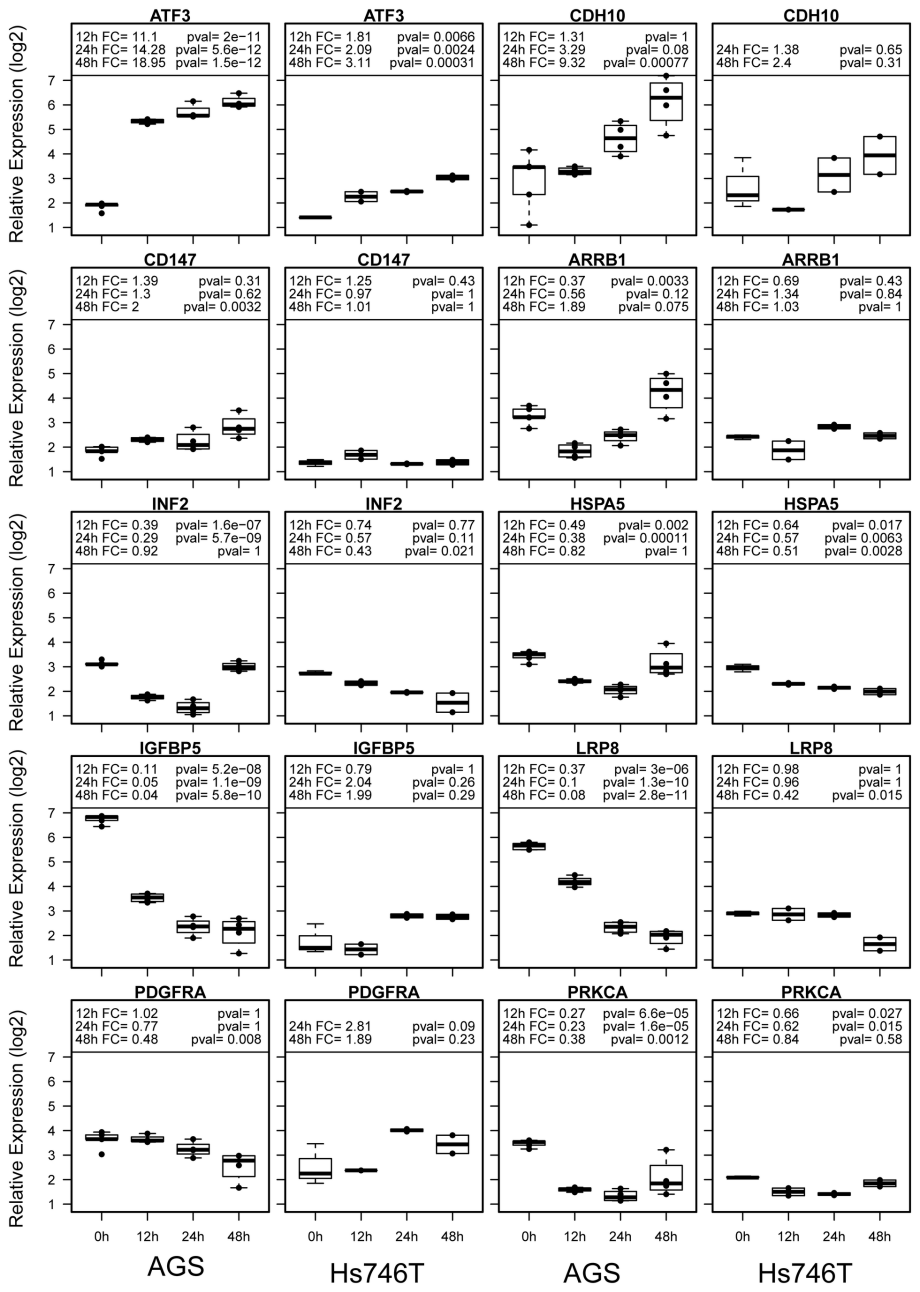
Confirmation of gene expression differences by real-time RT-PCR. Box blots are shown for each gene. Relative expression levels are shown in log 2 scale. FC (fold change) for each time point (12h, 24h and 48h) is compared to untreated controls (0h) with respective p-values.

Since the main focus of this study is to elucidate the molecular mechanism underlying MPA’s impact on cancer cell migration, we analyzed in detail the 50 migration-related genes with 2-4 fold differences and 15 migration-related genes with >4-fold differences between treated and untreated AGS cells (Figure 2). Four distinct clusters of genes are recognized. The cluster 1 genes are quickly up-regulated at the 12h time point and peaked at the 24h time point but slightly down-regulated later. The cluster 2 genes are slightly increased at the 24h time point but then become more up-regulated at the 48h and 72h time points. The cluster 3 genes are quickly down-regulated at the 12h and 24h time points but down-regulation becomes much less at later time points, while the cluster 4 genes are down-regulated at the 24h and later time points. The functional relationships among these genes are depicted in a gene regulatory network (Figure 3). Subsequently, we also assessed the validity of the expression data by analyzing a subset of candidate genes using real-time RT-PCR (Figure 4). A different set of RNA samples were obtained at different treatment time points (0, 12, 24 and 48 hours after treatment) from the MPA-sensitive AGS cells and MPA-insensitive Hs746T cells.

MPA treatment severely down-regulated two cell surface receptor genes (*PDGFRA* and *LRP8*), a protein kinase gene (*PRKCA*), and five other genes (*INF2*, *DOCK1*, *HSPA5*, *ARRB1*, and *IGFBP5*) (Figure 2). Six of these genes were selected for RT-PCR confirmation and all six genes were confirmed to be significantly down-regulated by MPA in AGS cells that are sensitive to MPA (Figure 4). Interestingly, most of these genes were not significantly down-regulated by MPA treatment in the Hs746T cells that are insensitive to MPA treatment (Figure 4).

Furthermore, MPA treatment of AGS cells significantly up-regulated the expression of a large number of genes implicated in cell migration. Seven genes (*ATF3*, *SMAD3*, *CITED2*, *CEAMCAM1*, *CYR61*, *NOS3* and *CDH10*) have more than four-fold difference and are highlighted in the microarray heatmap (Figure 2). Real-time RT-PCR confirmed that *ATF3* is increased by 10-20 folds in the AGS cells after MPA treatment, while it is only increased by 2-3 folds in the Hs746T cells (Figure 4). Similarly, MPA treatment significantly increased *CDH10* expression in AGS cells but not in Hs746T cells (Figure 4). 

We also performed pathway enrichment analysis on the 65 cell migration genes differentially expressed after MPA treatment. The top five KEGG pathways that are significantly altered by MPA are presented in Table 1. The focal adhesion, actin cytoskeleton and axon guidance pathways are highly related to cell migration and are significantly inhibited by MPA treatment, consistent with the observed reduction in the migration ability of MPA-treated AGS cells. Furthermore, the pathway in cancer is also significantly inhibited as several genes play important roles in cell proliferation and apoptosis. The TGF-beta pathway is significantly activated by MPA treatment and this finding is consistent with the suppression function of TGF-beta and MPA’s activity in inhibiting cell migration and proliferation. 

**Table 1:** Top five KEGG-pathways of the 65 cell migration genes significantly altered by MPA treatment.

**Pathway Name**	**ID**	**pFDR**	**Status**
Focal adhesion	4510	1.46E-06	Inhibited
Regulation of actin cytoskeleton	4810	1.22E-05	Inhibited
Pathways in cancer	5200	2.21E-05	Inhibited
Axon guidance	4360	0.00016	Inhibited
TGF-beta signaling pathway	4350	0.00021	Activated

### MPA-induced signaling changes related to migration

**Figure 5:**
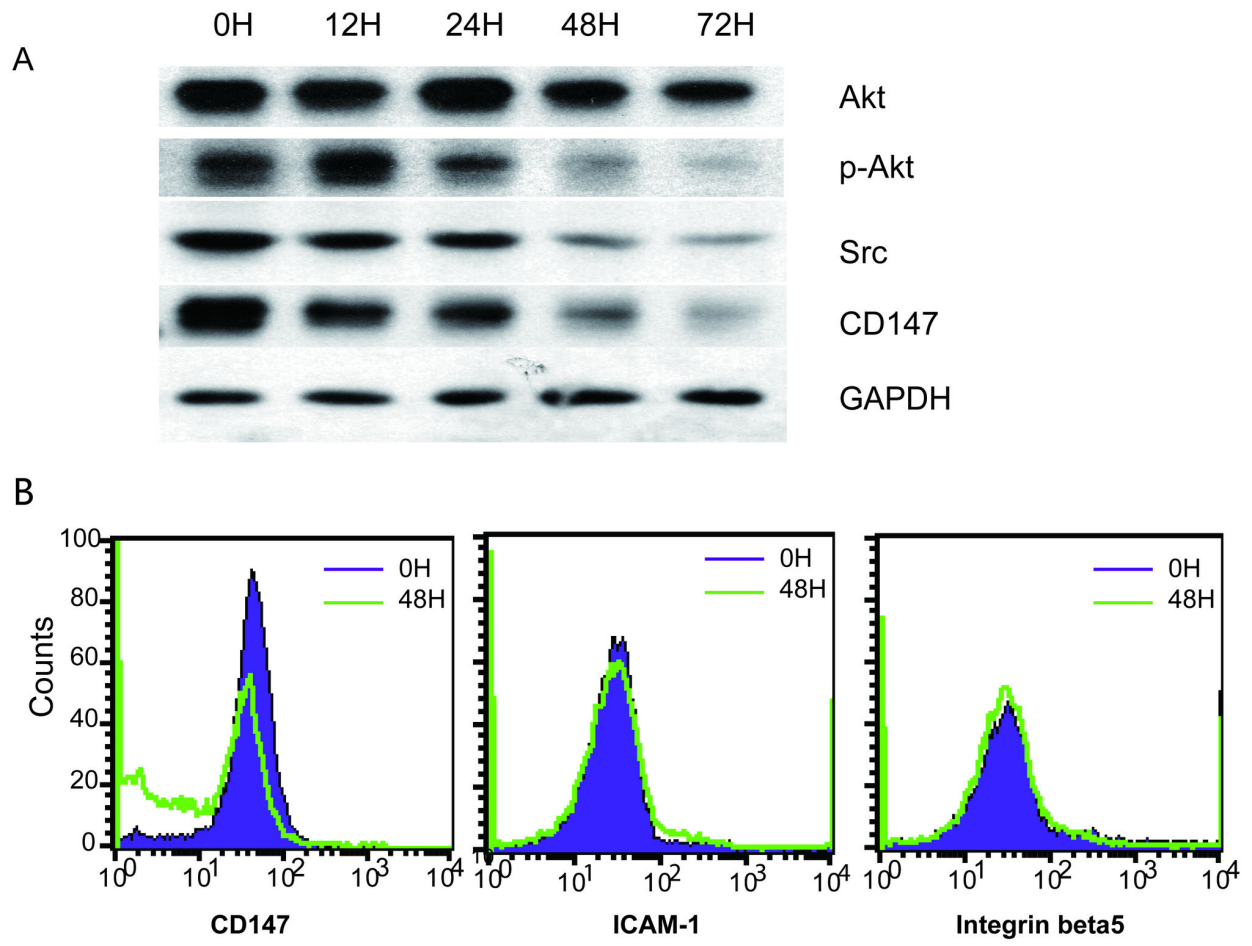
Proteomic changes in AGS cells after MPA treatment. **A**: Western blots for selected proteins. **B**: FACS analysis.

*AKT* is a key gene that connects with a large number of migration genes differentially expressed in the microarray data. However, the expression of the *AKT* genes was not altered by MPA treatment. Furthermore, the total AKT protein only showed slight change at later time points (Figure 5A). Interestingly, phospho-Akt (Ser473) was severely decreased by MPA treatment (Figure 5A). These results suggest that MPA may also alter the migration ability of AGS cell via influence on the activation status of key signaling proteins such as AKT, in addition to regulating the expression of genes.

The expression of the genes encoding the Src tyrosine kinase is only marginally down-regulated in the microarray data. Since Src is functionally related to a large number of migration genes differentially expressed in the microarray data (Figure 3), we determined the protein expression level of Src and found that the protein is drastically down-regulated by MPA treatment (Figure 5A). However, the precise reason for the observed gene and protein expression discrepancies is unclear and remains to be further investigated. 

### MPA-induced CD147 reduction

The Src tyrosine kinase also transmits integrin-dependent signals central to cell movement and proliferation. Several differential expressed genes (*CEACAM1*and *CYR61*) are also implicated in the integrin signaling pathway. Therefore, we analyzed the expression of several integrins on AGS and Hs746T cell surface using FACS analysis. Although the expression of the *CD147* gene was not altered by MPA treatment (Figure 4), the CD147 surface protein expression was dramatically down regulated after MPA treatment (Figure 5B). Furthermore, Western blot analysis also confirmed that the total CD147 protein was significantly down-regulated by MPA treatment (Figure 5A). Interestingly, MPA treatment of Hs746T cells did not significantly alter CD147 surface expression. The expression of ICAM-1 and integrin beta 5 was not changed by MPA treatment (Figure 5B).

### MPA reduces matrix metalloproteinase 1 (MMP-1)

**Figure 6:**
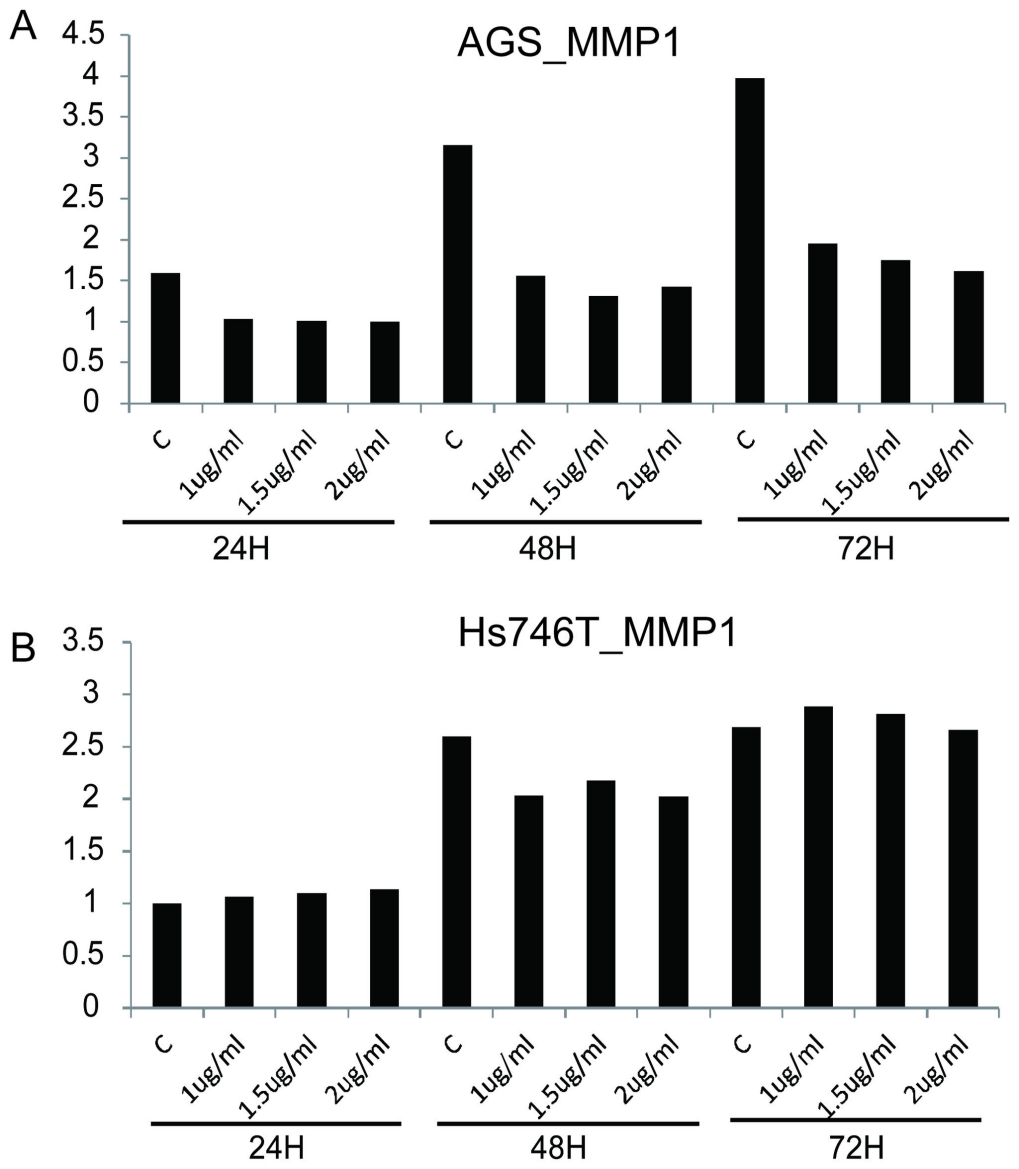
MMP1 protein levels in the cell culture medium. **A**: AGS cells. **B**: Hs746T cells.

Three MMP proteins were measured in the culture medium of AGS and Hs746T cells after MPA treatment using Luminex assays (Figure 6). The protein levels of MMP2 and MMP9 were very low in AGS cell culture medium (data not shown). MMP1 was significantly reduced by MPA treatment in a dose-dependent manner in AGS cell culture medium (Figure 6A), while no significant change was observed in Hs746T cells (Figure 6B). 

## Discussion

MPA is known to specifically inhibit IMPDH, the rate-limiting enzyme for the *de novo* synthesis of guanosine nucleotides, which are essential for DNA replication as well as RNA and protein synthesis. MPA is also well known to inhibit cancer cell proliferation and induce apoptosis. In this study, we demonstrated for the first time that MPA can significantly reduce the migration and invasion abilities of AGS cells. Since metastasis is a major problem facing cancer patients, MPA’s ability to inhibit cancer cell migration and invasion, in addition to its ability to inhibit cell proliferation and induce apoptosis, renders MPA an excellent anti-cancer drug candidate.

In this study we also used a variety of molecular techniques to elucidate the molecular mechanisms underlying MPA’s function to inhibit cancer cell migration. We first used global transcriptomic analyses to identify migration candidate genes altered by MPA treatment. These studies suggested 50 genes with 2-4 fold differences and 15 genes with > 4 fold differences (Figure 2). These genes include cell surface receptors, protein kinases, and other genes involved in the regulation of migration. The validity of the microarray data has been confirmed by real-time RT-PCR for selected genes with > 4-fold differences (Figure 4). In addition to transcriptomic analysis, we also used several proteomic and functional technologies to elucidate the molecular mechanisms underlying the anti-migration activity of MPA. Transcriptomic profiling is a powerful technology to assess global molecular changes. The major advantage of gene expression microarray is its ability to rapidly and economically evaluate the expression pattern of almost all genes expressed in the cells of interest. Despite the power of this class of technologies, there are a number of issues that limit microarray primarily as a hypotheses-generating tool. The most serious limitation is the imperfect correlation or lack of correlation between gene expression and protein expression/function. This deficiency is well illustrated in this study as major differences in protein expression of activation status were altered by MPA while the gene expression levels were unchanged. Therefore, it is essential to combine transcriptomic and proteomic technologies for global characterization of biological functions.

At least three genes that are known to promote cell migration (*INF2*, *DOCK1*, and *HSPA5*) are severely down-regulated (>4-fold) by MPA treatment (Figure 2). *INF2* encodes the inverted formin 2 protein, which promotes the formation of detyrosinated microtubules necessary for centromere reorientation in migrating cells [25]. The dedicator of cytokinesis 1 (DOCK1) protein interacts with HER2 and promotes HER2-induced Rac activation and cell migration [26]. HSPA5 is a stress response protein induced by agents or conditions that adversely affect endoplasmic reticulum function. It actively regulates multiple malignant phenotypes including cell growth, migration, and invasion.  Down-regulation of these genes by MPA is consistent with the reduced migratory capacity of AGS cells after MPA treatment. In contrast, overexpression of β-arrestin-1 (*ARRB1*) reduced the migratory propensity of breast cancer cells lines, whereas silencing increased migration [27]. In a more recent study [28], *ARRB1* was found to promote the migratory ability of lung cancer migration. Therefore, it is unclear at this time whether *ARRB1* inhibits or promotes migration in AGS cells. Another drastically down-regulated gene, *IGFBP5*, induces cell adhesion and increases cell survival in MCF-7 human breast cancer cells [29]. IGFBP5 seems to inhibit migration of MCF7 cells [29] but promotes migration of peripheral blood mononuclear cells [30]. Therefore, the role of IGFBP5 on AGS cell migration remains to be determined. 

MPA treatment of AGS cells also significantly up-regulated the expression of a number of genes implicated in cell migration. Among these, seven genes (*ATF3*, *SMAD3*, *CITED2*, *CEAMCAM1*, *CYR61*, *NOS3* and *CDH10*) are of great interest as they show more than four-fold differences in the microarray heatmap (Figure 2). Two of the seven genes (*ATF3* and *CDH10*) were selected and confirmed by real-time RT-PCR. *ATF3* expression is increased by 11-fold 12 hours after MPA treatment and by 19-fold at 48 hours. *CDH10* expression is altered in AGS cells but not in Hs746T cells (Figure 4). ATF3 has recently been shown to suppress metastasis of bladder cancer through regulating Gelson-mediated remodeling of the actin cytoskeleton. Overexpression of *ATF3* in highly metastatic bladder cancer cells decreases migration *in vitro* and *in vivo*[31]. The TGF-β/Smad signaling pathway plays a critical role in pancreatic ductal adenocarcinoma growth, migration and metastasis. SiRNA-mediated silencing of SMAD3 increases TGF-β-induced cancer cell migration [32]. Consistent with the reduced migratory ability of AGS cells after MPA treatment, SMAD3 is significantly up-regulated by MPA treatment (Figure 2). *CITED2* encodes the Cbp/p300-interacting transactivator 2 protein, which positively regulates TGF-β signaling through its association with the SMAD/p300/CBP-mediated transcriptional coactivator complex and stimulates several other transcriptional activities. It has been shown that *Cited2* knockout mice have increased gonadal cell migration [33]. Therefore, up-regulated *CITED2* gene expression may contribute to the reduced migratory ability of AGS cells treated with MPA. *CEACAM1* encodes the carcinoembryonic antigen-related cell adhesion molecule (CD66a), which affects integrin-dependent signaling and regulates extracellular matrix protein-specific morphology and migration of endothelial cells [34]. Although most studies have shown that CEACAM1 alone plays a promigratory role, the interaction of CEACAM1 and filamin A drastically reduced migration and cell scattering [35]. Therefore, the role of CEACAM1 in cell migration may depend on the cellular context.

However, two of the genes highly up-regulated by MPA (*CYR61* and *NOS3*) actually promote migration. *CYR61* encodes the cysteine-rich protein 61, which promotes cell migration via alteration of the functions of integrin [36]. The up-regulation of *CYR61* by MPA treatment may reduce the anti-migratory role of MPA. Nitric oxide promotes mammary tumor cell migration through sequential activation of nitric oxide synthase, guanylate cyclase and mitogen-activated protein kinase [37]. *NOS3* encodes the nitric oxide synthase 3 and is highly up-regulated by MPA treatment. Again, up-regulation of *NOS3* may reduce the antimigratory function of MPA. The *CDH10* gene encodes one of the cadherin proteins that play critical roles in cell-cell adhesion. It has been inferred that CDH10 may play a critical role in mesendodermal cell migration although this inferred relationship has not been experimentally tested. *CDH10* is drastically increased by MPA treatment of AGS cells but not in Hs746T cells and its role in migration should be experimentally tested. 

*PDGFRA* encodes a cell surface tyrosine kinase receptor member of the platelet-derived growth factor family and is known to be closely related with cell migration [38]. Low-density lipoprotein receptor-related protein 8 (LRP8), also known as apolipoprotein E receptor 2 (ApoER2), is a cell surface receptor with an EGF-like domain. These receptors function in signal transduction and endocytosis of specific ligands. LRP8 is known to play an important role in embryonic neuronal migration [39]. Extracellular ligand binding to LRP8 and a related receptor, VLDLR, on migrating neurons activates intracellular processes that begin with Dab1 phosphorylation [39]. Dab1, a tyrosine kinase phosphorylated protein, can be phosphorylated by two tyrosine kinases (Src and Fyn) and phosphorylated Dab1 induces further activation of these two kinases and other kinases including PI3K. Phosphorylated PI3K may lead to inhibitory phosphorylation of the tau kinase glycogen synthase kinase 3 beta (GSK3B), which alters the activity of tau protein that is involved in microtubule stabilization. PI3K can also phosphorylate protein kinase C proteins (PKC), which phosphorylate a wide variety of protein targets and are known to be involved in diverse cellular signaling pathways. PKC-alpha, encoded by the *PRKCA* gene, is a member of the PKC family and has been implicated in cancer cell migration [40,41]. MPA treatment of AGS cells significantly down-regulates this entire pathway including *LRP8* and *PRKCA* gene expression (Figure 2 & 4), consequently leading to reduced cell migration.

 The Src family of non-receptor tyrosine kinases has been implicated in the intracellular signaling cascade that acts downstream of cell surface receptors to elicit different cellular functions including growth, proliferation, adhesion, motility and angiogenesis[42]. Src tyrosine kinases also transmit integrin-dependent signals central to cell movement and proliferation. Hallmarks of v-src induced transformation are rounding of the cell and the formation of actin rich podosomes, which are correlated with increased invasiveness, a process essential for metastasis. As the expression of the Src protein is drastically down-regulated by MPA treatment, our results suggest that the Src signaling pathway may play important roles in modulating cancer migration by MPA treatment. 

AKT is a serine/threonine-specific protein kinase that plays a key role in multiple cellular processes such as cell proliferation, apoptosis, transcription and cell migration[43,44]. The expression of the AKT genes was not altered by MPA treatment and the total AKT protein only showed slight change at later time points. AKT can be phosphorylated by its activating kinases, phosphoinositide dependent kinase 1 and the mammalian target of rapamycin complex 2 (mTORC2). Activated AKT can then go on to activate or deactivate its myriad substrates (mTOR) via its kinase activity. We have shown in this study that MPA treatment can severely decrease phospho-Akt (Ser473) thus reducing the AKT activity. Reduced AKT activity may be a major pathway leading to reduced migration ability of the AGS cells. Furthermore, reduced AKT activity is also consistent with the reduced proliferation capacity of the MPA-treated AGS cells. 

As a number of the genes identified by our microarray data are implicated in integrin signaling, we investigated the expression of three integrins after MPA treatment. Indeed, we observed significant reduction of CD147 after MPA treatment. CD147 plays multifunctional roles in tumor progression[45]. CD147 induces angiogenesis by stimulating VEGF production, multidrug resistance through up-regulation of ErbB2 signaling, and invasiveness via stimulation of matrix metalloproteinase production. The MPA-induced down-regulation of CD147 is consistent with our observation that MMP1 expression is down-regulated by MPA treatment. MMPs are involved in the breakdown of extracellular matrix in normal physiological processes, such as embryonic development, reproduction, and tissue remodeling, as well as in disease processes, such as arthritis and metastasis. MMPs break down the interstitial collagens and can increase the migration and invasion ability [46–48]. The reduction of MMPs may be an important mechanism through which MPA inhibits cancer cell migration and invasion. 

In conclusion, we demonstrated that MPA treatment can significantly inhibit AGS migration and invasion. Our molecular studies using a variety of techniques further indicate that MPA modulates cancer cell migration through down-regulation of a large number of genes with promigratory functions (*PRKCA*, *DOCK1*, *INF2, HSPA5, LRP8* and *PDGFRA*) and proteins (pAKT, Src, CD147 and MMP1) as well as up-regulation of a number of genes with anti-migratory functions (*ATF3*, *SMAD3*, *CITED2* and *CEAMCAM1*). However, a few genes that can promote migration (*CYR61* and *NOS3*) were up-regulated. Among these important genes implicated in cell migration, 15 genes are consistent and 2 genes are inconsistent with the observed reduction of AGS cell migration after MPA treatment. Therefore, MPA’s overall antimigratory role on cancer cells reflects a balance between promigratory and antimigratory signals influenced by MPA treatment.
